# Effect of WEDM Process Parameters on Surface Morphology of Nitinol Shape Memory Alloy

**DOI:** 10.3390/ma13214943

**Published:** 2020-11-03

**Authors:** Rakesh Chaudhari, Jay J. Vora, Vivek Patel, L. N. López de Lacalle, D. M. Parikh

**Affiliations:** 1 Department of Mechanical Engineering, School of Technology, Pandit Deendayal Petroleum University, Raisan, Gandhinagar 382007, India; chaudharirakesh5@gmail.com (R.C.); vorajaykumar@gmail.com (J.J.V.); dm.parikh@sot.pdpu.ac.in (D.M.P.); 2 School of Material Science and Engineering, Northwestern Polytechnical University, Shaanxi 710072, China; profvvp@yahoo.com; 3 Department of Engineering Science, Division of Welding Technology, University West, 46186 Trollhättan, Sweden; 4 Department of Mechanical Engineering, University of the Basque Country, Escuela Superior de Ingenieros Alameda de Urquijo s/n., 48013 Bilbao, Spain

**Keywords:** shape memory alloy, nitinol, WEDM, shape memory effect, surface morphology, molybdenum tool wire

## Abstract

Nickel–titanium shape memory alloys (SMAs) have started becoming popular owing to their unique ability to memorize or regain their original shape from the plastically deformed condition by means of heating or magnetic or mechanical loading. Nickel–titanium alloys, commonly known as nitinol, have been widely used in actuators, microelectromechanical system (MEMS) devices, and many other applications, including in the biomedical, aerospace, and automotive fields. However, nitinol is a difficult-to-cut material because of its versatile specific properties such as the shape memory effect, superelasticity, high specific strength, high wear and corrosion resistance, and severe strain hardening. There are several challenges faced when machining nitinol SMA with conventional machining techniques. Noncontact operation of the wire electrical discharge machining (WEDM) process between the tool (wire) and workpiece significantly eliminates the problems of conventional machining processes. The WEDM process consists of multiple input parameters that should be controlled to obtain great surface quality. In this study, the effect of WEDM process parameters on the surface morphology of nitinol SMA was studied using 3D surface analysis, scanning electron microscopy (SEM), and energy-dispersive X-ray (EDX) analysis. 3D surface analysis results indicated a higher value of surface roughness (SR) on the top of the work surface and a lower SR on the bottom portion of the work surface. The surface morphology of the machined sample obtained at optimized parameters showed a reduction in microcracks, micropores, and globules in comparison with the machined surface obtained at a high discharge energy level. EDX analysis indicated a machined surface free of molybdenum (tool electrode).

## 1. Introduction

In the present scenario of manufacturing competitiveness, the adoption of new technologies is essential to overcome the challenges of achieving component accuracy, high quality, acceptable surface finish, increased production rate, enhanced product life, and reduced environmental impact. Beyond these conventional challenges, the machining of newly developed smart materials also requires inputs of intelligent machining strategies. For the manufacturing processes, the accuracy of the finished product is mainly dependent on its input process parameters. Therefore, it is necessary to control the input parameters and secure their optimum values. One such newly developed generation of alloys is the shape memory alloys (SMAs). SMAs display an exceptional characteristic of regaining their shape when they are heated. Nickel–titanium alloy is one of the shape memory alloys, which is known as nitinol in respect of its innovation at the Naval Ordnance Laboratory (NOL) [1]. Shape memory alloys were initially revealed by Swedish physicist Arne Olander [2]. He found that Au–Cd alloys returned to their initial shape and size after heating above a certain temperature even after their plastic deformation in the cold state. Out of numbers of combinations of SMAs, nitinol became a famous shape memory alloy owing to low production cost as compared to other SMAs, safer and easier handling, and superior mechanical properties. Nitinol SMAs have been applied in various areas like air conditioning vents, electronic cable connectors, and valves [3]. Over the last decade, the areas of application also spread to aerospace, oil industries, automobiles, and robotics. These smart materials possess the main characteristics of superelasticity (SE) and shape memory effect (SME) [1]. Nitinol is considered an ideal material in the biomedical field owing to its properties like biocompatibility and wear and corrosion resistance [4,5]. Due to the functional properties of nitinol SMAs, their biomedical application has proven successful by increasing the possibility as well as the performance quality of minimally invasive surgeries. The nickel–titanium SMA is highly biocompatible, which makes it useful in orthopedic implants, surgical instruments, cardiovascular devices, and orthodontic devices [6,7]. Nitinol SMA is categorized into two different types, namely shape memory and superelastic. If the temperature of shape recovery is less than the room temperature, it is recognized as superelastic; if the temperature of the shape recovery is greater than room temperature, it is recognized as shape memory [8]. There are several challenges faced when using conventional machining techniques to machine nitinol SMA; these challenges are due to severe strain hardening, high ductility, poor chip breaking, superelasticity, burr formation, and high wear and corrosion resistance [9,10]. Weinert and Petzoldt concluded that the machining of NiTi-based alloys is complex using conventional techniques like turning, drilling, and deep hole drilling. Poor chip breaking, tool wear, and burr formation have been observed while machining shape memory alloys using conventional machining techniques

Wire electrical discharge machining (WEDM) is a type of nonconventional machining method which is more preferable to overcome these defects [10]. The WEDM process is applicable to all conductive materials regardless of material hardness [11,12,13,14]. The WEDM process creates a series of sparks that helps to remove the material from the work surface. These sparks are generated between the wire (electrode) and the workpiece in the presence of a dielectric fluid. The WEDM process consists of a high number of process parameters that should be controlled to acquire great surface quality and control the resulting metallurgical and mechanical properties. Achieving superior performance of nitinol using the WEDM process requires the optimized parameter settings of input process parameters such as discharge current (I), pulse-off time (T_on_), pulse-on time (T_off_), and dielectric fluid pressure for machining of nitinol SMA [15]. Chaudhari et al. [10] identified current (I), T_on_, and T_off_ as key input parameters for machining of superelastic SMA. Their results indicated that all the parameters are significant for achieving simultaneous output parameters. In another study, Majumder and Maity [16] used the WEDM technique for the simultaneous optimization of multiple input parameters of nitinol SMA. Soni et al. [17] identified the appropriate level of pulse-on time and servo voltage for the reduction of microcracks. Proper selection of input process parameters is a key factor in the WEDM process to avoid wire rupture problems and the formation of larger craters on the work surface [18]. In past research, electrodes of brass and copper wires have been used for surface analysis of SMAs for samples machined by WEDM [19,20]. 

Recently, we conducted a Pareto analysis of WEDM input parameters for machining of nitinol SMA using the heat transfer search (HTS) algorithm [21]. The HTS algorithm was found to be effective for predicting and optimizing the input values for all objective functions. ANOVA test results showed the robustness of the generated empirical models for the optimization of multiple responses. The key focus of the present study is on the influence of input parameters on the surface integrity of the machined surface. Based on reviewed literature and machining capabilities, current (I), pulse-off time (T_off_), and pulse-on time (T_on_) were selected as three key input parameters surface roughness (SR), material removal rate (MRR), and microhardness (MH) were selected as output characteristics. For surface morphology, sets of parameters with high and low discharge energy levels and a set of optimized process parameters were considered. In past studies, more focus was placed on the parametric optimization of nitinol alloys. However, not much has been reported on surface integrity after conducting parametric optimization. In the present study, scanning electron microscope (SEM) and energy-dispersive X-ray analysis (EDX) were used to study elemental composition, surface analysis, and phase analysis after the machining of nitinol SMA. The aim of the present study was to provide significant input to end-users for the selection of WEDM process parameters for nitinol SMA.

## 2. Materials and Methods 

In the current study, a Concord WEDM machine DK7732 with EDM oil (Concord Limited, Bangalore, India) as dielectric fluid was used to machine the samples of nitinol SMA (Procured from SMA Wires, Ahmedabad, India). The chemical composition of nitinol SMA (Ni_55.8_Ti) used in the present study is shown in Table 1. A nitinol bar with a diameter of 6 mm was utilized to machine tests for surface investigation. In a past report, the impact of input parameters was inspected by cutting sample sizes of 1.5 mm. A similar size of 1.5 mm was favored in the current examination. A reusable molybdenum wire (Concord Limited, Bangalore, India) of 0.18 mm diameter was used as a tool electrode. 

**Figure 1:**
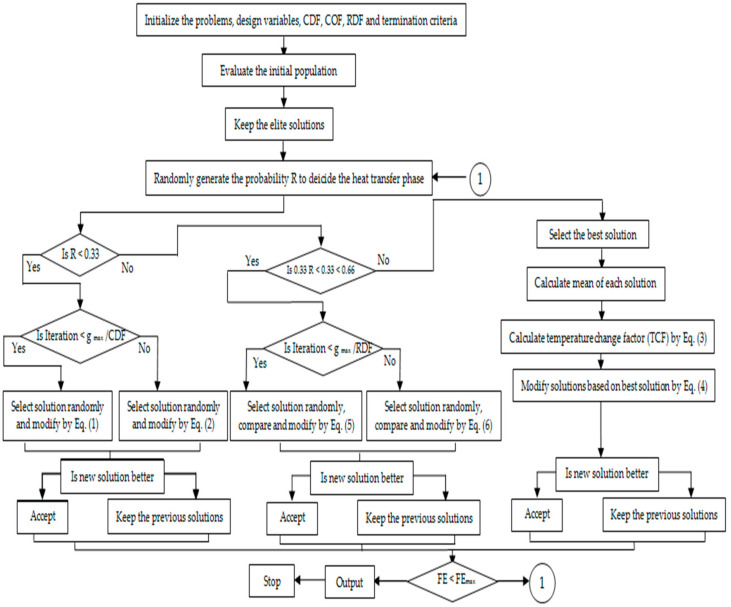
Flow chart of the heat transfer search (HTS) algorithm.

The heat transfer search (HTS) technique has been used for multiobjective optimization of multiple variables like MRR, SR, and MH [10,21]. The HTS algorithm functions on basis of the transfer of heat owing to the interface between the system particles and the surroundings to achieve thermal stability. Thermal stability in the transfer of heat between system and surroundings can be obtained from a thermodynamically imbalanced system. To reach thermal stability, three heat transfer phenomena, namely conduction, convection, and radiation, contribute to a large extent. As such, the three phases of conduction, convection, and radiation are considered during the implementation of the HTS algorithm. During the implementation of the HTS algorithm, each heat transfer phenomenon has the same opportunity for heat transfer, and each generation decides any one of these three phenomena randomly. Arbitrarily created population commences the HTS algorithm, in which the system consists of ‘n’ number of particles (population size) and temperature level (input variables). Subsequently, arbitrarily chosen heat transfer phenomena update the population size for each generation. In the next stage, an updated solution having a good functional rate gets accepted and the worst solution gets replaced by elite solutions [10]. The flow chart of the HTS algorithm is shown in Figure 1. 

### 2.1. Conduction Phase

Equations (1) and (2) are used to update the solutions for the conduction phase:

(1) Xj,i’=Xk,i+−R2Xk,i, iffXj>fXkXj,i+−R2Xj,i, iffXj<fXk;ifg≤gmaxCDF

(2) Xj,i’=Xk,i+−riXk,i, iffXj>fXkXj,i+−riXj,i, iffXj<fXk;ifg>gmaxCDF

where $X_{j,i}^{’}$ is the updated solution; j = 1, 2, …, n; k is a randomly selected solution; j ≠ k; k ∈ (1, 2, …, n); i is a randomly selected design variable; i ∈ (1, 2, …, m); g_max_ is the maximum number of generation specified; CDF is the conduction factor; R is the probability variable; R ∈ {0, 0.3333}; and r_i_ ∈ {0, 1} is a uniformly distributed random number [10].

### 2.2. Convection Phase

Equations (3) and (4) are used to update the solutions for convection phase:

(3) $$X_{j,i}^{’}=X_{j,i}+R\times \left(X_{s}-X_{\mathrm{ms}}\times \mathrm{TCF}\right)$$

(4) TCF=absR−ri, ifg≤gmaxCOFround1+ri, ifg>gmaxCOF

where $X_{j,i}^{’}\;$is the updated solution; j = 1, 2, …, n; i = 1, 2, …, m; COF is the convection factor; R is the probability variable; R ∈ {0.6666, 1}; r_i_ ∈ {0, 1} is a uniformly distributed random number; X_s_ is the temperature of the surroundings; X_ms_ is the mean temperature of the system; and TCF is a temperature change factor [10]. 

### 2.3. Radiation Phase

Equations (5) and (6) are used to update the solutions for radiation phase:

(5) Xj,i’=Xj,i+R×Xk,i−Xj,i, iffXj>fXkXj,i+R×Xj,i−Xk,i, iffXj<fXk ;ifg≤gmaxRDF

(6) Xj,i’=Xj,i+ri×Xk,i−Xj,i, iffXj>fXkXj,i+ri×Xj,i−Xk,i, iffXj<fXk ;ifg>gmaxRDF

where $X_{j,i}^{’}\;$is the updated solution; j = 1, 2, …, n; i = 1, 2, …, m; j ≠ k; k ∈ (1, 2, …, n), and k is a randomly selected molecule; RDF is the radiation factor; R is the probability variable; R ∈ {0.3333, 0.6666}; and r_i_ ∈ {0, 1} is a uniformly distributed random number [10]. 

It is not possible to cover the entire machining range in the design of the experiment. Based on the literature, suitable ranges for input process parameters were considered while conducting the experiments as follows:

- Pulse on time: 35 µs ≤ T_on_ ≤ 55 µs;
- Pulse off time: 10 µs ≤ T_off_ ≤ 20 µs;
- Current: 2 A ≤ I ≤ 4 A.

However, there is a possibility that the optimized parameter settings may be outside these considered ranges. For this, the machining range was considered between the extreme limits of input process parameters while implementing the HTS algorithm as follows:

- Pulse on time: 1 µs ≤ T_on_ ≤ 110 µs;
- Pulse off time: 1 µs ≤ T_off_ ≤ 32 µs;
- Current: 1 A ≤ I ≤ 6 A.

**Figure 2:**
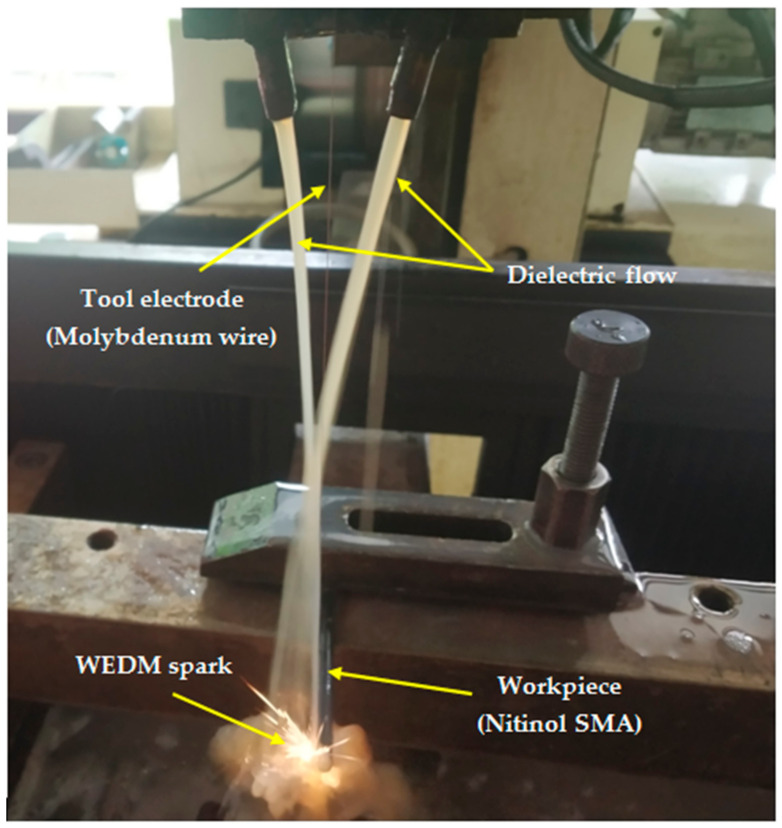
Flushing mechanism for the wire electrical discharge machining (WEDM) process.

The optimum values for selected responses were found at T_on_ of 40 μs, T_off_ of 12 μs, and current of 1 A [21]. For these optimal conditions, the responses of other output variables were predicted, and a validation test was performed using these predicted process parameters. The HTS algorithm was found to be capable of successfully predicting and optimizing the process parameters as the difference between the predicted and measured value was negligible. In the present study, the effect of input process parameters at these optimum levels was studied in detail on the surface integrity of the machined surface. Figure 2 shows the slide-flushing mechanism (from above) used in the current study during the WEDM process. To understand the variation of SR in different areas of the machined surface, a three-dimensional (3D) digital microscope with a noncontact-type probe (Keyence VHX-600, China) was used. Keyence VHX-600 digital microscope (Keyence VHX-600, China) was used to record the variation of SR values. The recorded SR values in the current study have an arithmetic average roughness (Ra) in µm. The surface morphology of the machined samples was examined using SEM and EDX equipment from Tescan (Vega Tescan, India). Etchant (82 mL H_2_O + 14 mL HNO_3_ + 4 mL HF) was used for nitinol SMA. 

## 3. Results and Discussion

### 3.1. Analysis of SR Using 3D Digital Microscope 

**Figure 3:**
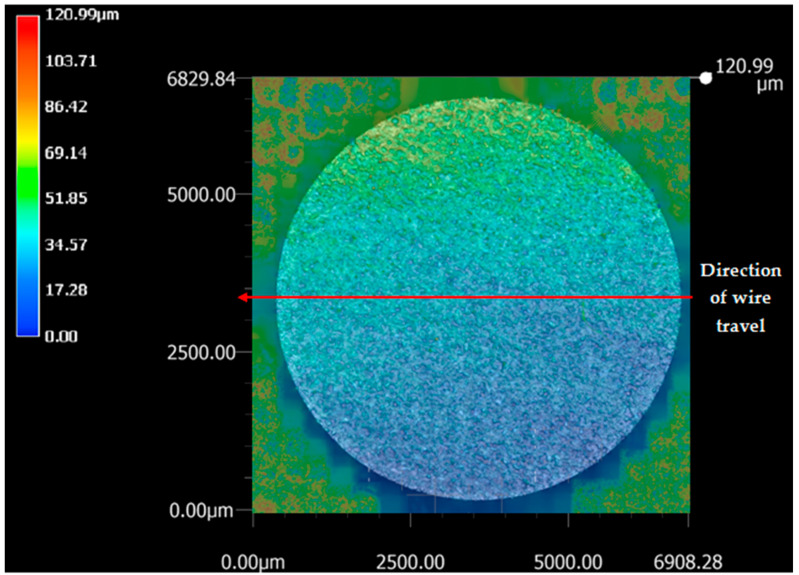
Surface machined under optimized process parameters.

**Figure 4:**
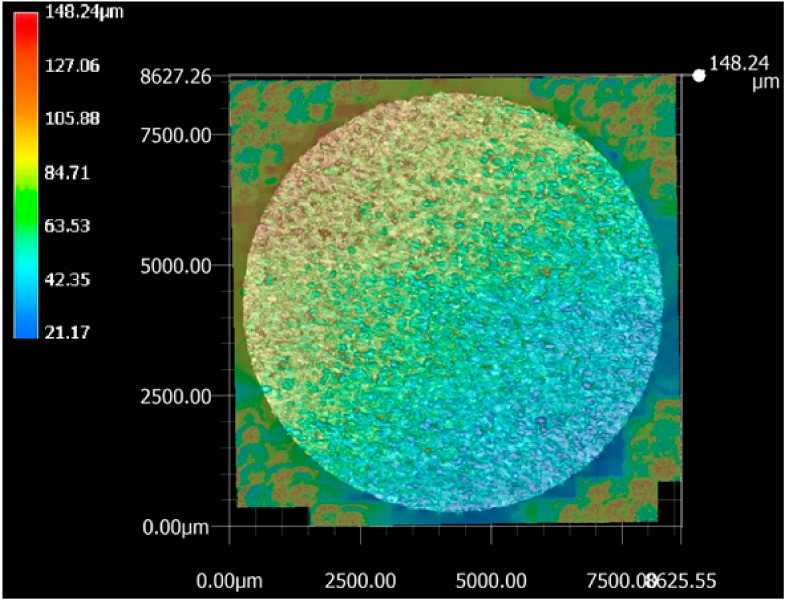
Machined surface representing surface roughness (SR) values for superelastic nitinol [22].

The sample machined using the optimized set of parameters (T_on_ of 40 μs, T_off_ of 12 μs, and current of 1 A) was subjected to 3D analysis. Differential scanning calorimetry (DSC) (Netzsch, Selb, Germany) testing revealed that the WEDM sample machined using the optimized set of parameters had retention of shape memory effect when compared to that of the starting base material [21]. Figure 3 shows the top surface of the machined sample, with SR values indicated at various locations. The top surface of the machined sample shows the highest value of SR, which is indicated by green color; the bottom surface of the machined sample shows the lowest value of SR, as indicated by blue color. While red color indicates the highest SR in the image, these points were not taken into account as they are outside of the machined surface. As indicated by the wire travel direction shown in Figure 3, SR was measured on the surface from left to right. Figure 4 shows the machined surface of superelastic nitinol SMA, with SR values represented. It can be clearly observed that the average value of SR in the current study (Figure 3) is less than the average value of SR obtained for superelastic nitinol shape memory alloy (Figure 4) [22]. 

**Figure 5:**
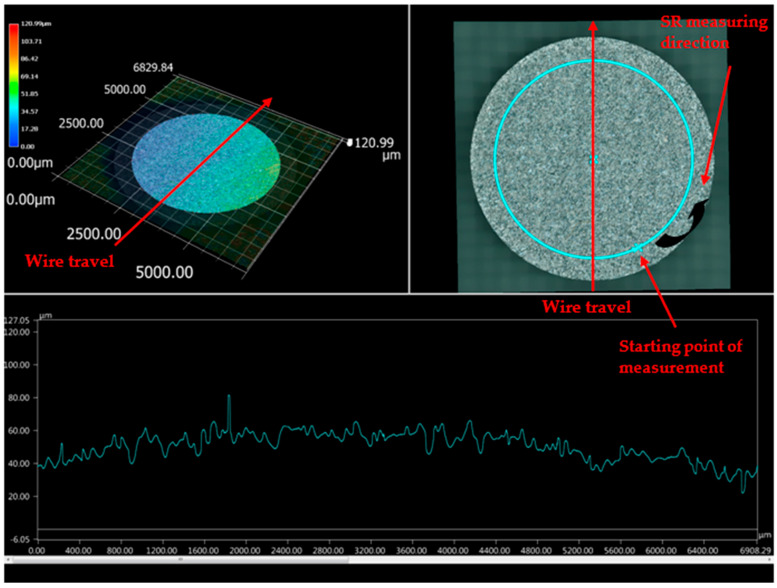
Surface analysis of machined sample at outermost periphery.

**Figure 6:**
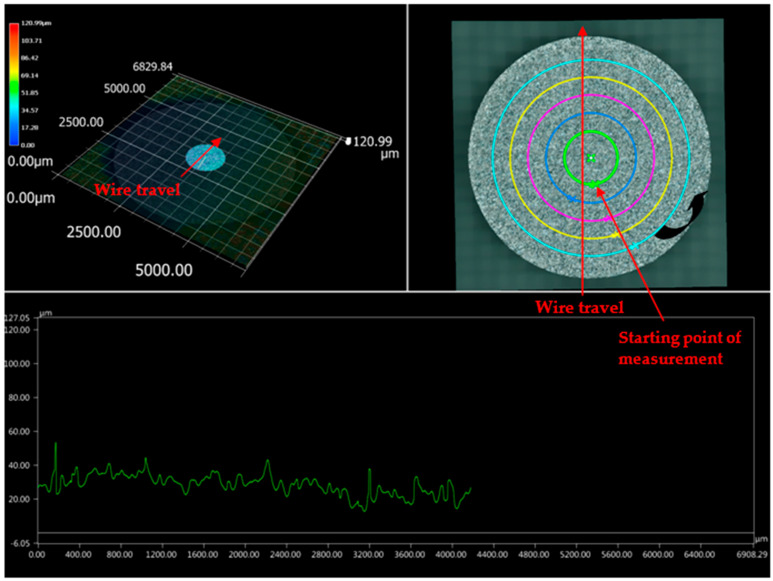
Surface analysis of machined sample at innermost periphery.

The dielectric flushing mechanism from above was the main reason for obtaining a higher value of SR on the top surface of the sample; i.e., a greater area of the top surface is exposed to the dielectric fluid. This in turn increases the discharge energy, which results in a greater amount of debris removal. It also increases the material removal rate due to the higher value of discharge energy. However, at the bottom surface of the sample, the supply of dielectric fluid is lower, which minimizes the discharge energy and SR of the bottom region. An increase in dielectric fluid pressure increases the discharge energy which increases the SR and forms the larger craters on the surface of the workpiece [23]. Figure 5 and Figure 6 support this conclusion of differences in SR between different locations of the work surface. Figure 5 and Figure 6 represent the detailed SR analysis of the machined sample at the outermost and innermost peripheries, respectively. The number of data points for each periphery is different due to different circumferential lengths for both peripheries. A distance of 0.43 mm was taken into account between the two consecutive SR measurements. The outermost periphery has a circumferential length of 20,920 µm with 48,651 data points, whereas the innermost periphery has a circumferential length of 8345 µm with 19,406 data points. The lowest value of SR was obtained at the bottom surface. while the maximum SR was observed to be at the top surface. However, it is recommended to have identical flushing pressure coming from both directions (above and below) of the workpiece to avoid any variation in SR value on the machined surface. This might result in giving a higher average SR value for the machined surface.

Wire rupture occurs with an increase in crater size. To avoid this problem, discharge energy must be at an optimum level. A lower SR value can be achieved at a lower discharge energy level. Lowering the values of pulse-on time and current results in a lower discharge energy level [24]. However, a lower value of pulse-off time gives a higher discharge energy level as it minimizes the time between two consecutive sparks. Pursuant to the same, lower discharge energy can be obtained at lower values of pulse-on time and current and a higher value of pulse-off time. The machined sample considered in the current study was obtained at optimized parameter settings (T_on_ of 40 μs, T_off_ of 12 μs, and current of 1 A). These values show that discharge energy was not at a lower or higher level as these values are derived for multiple response variables such as MRR, SR, and MH. Table 2 shows the higher discharge energy level of an optimized set of parameters in our previously reported study of the surface analysis of superelastic SMA in comparison with the current study [22]. Lower discharge energy at optimized parameter settings in the current study gives lower SR values as compared to the previously reported study, without compromising the optimum values of other response variables such as MRR and MH. The average value of SR in the current study is less than the average value obtained for superelastic nitinol shape memory alloy [22]. This is because of the lower discharge energy for parametric settings of nitinol SMA as compared to the discharge energy of superelastic nitinol shape memory alloy. 

### 3.2. Analysis of Surface Defects 

**Figure 7:**
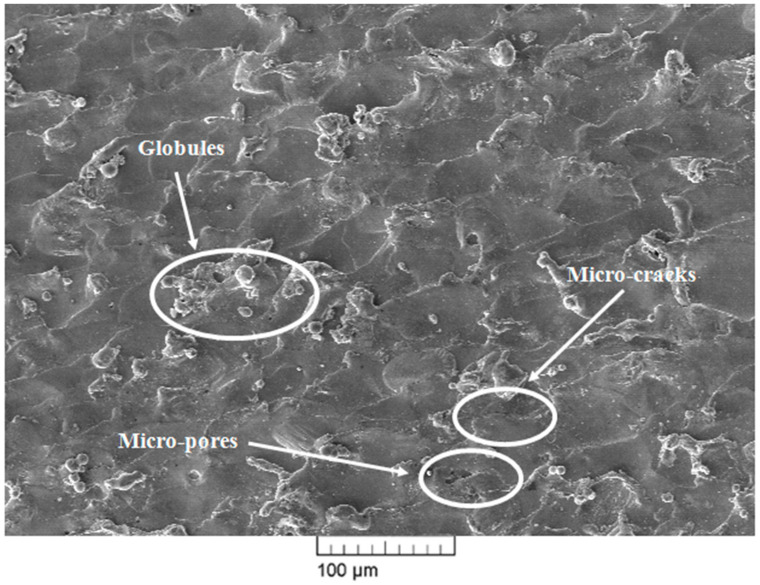
SEM micrograph at low discharge energy level.

**Figure 8:**
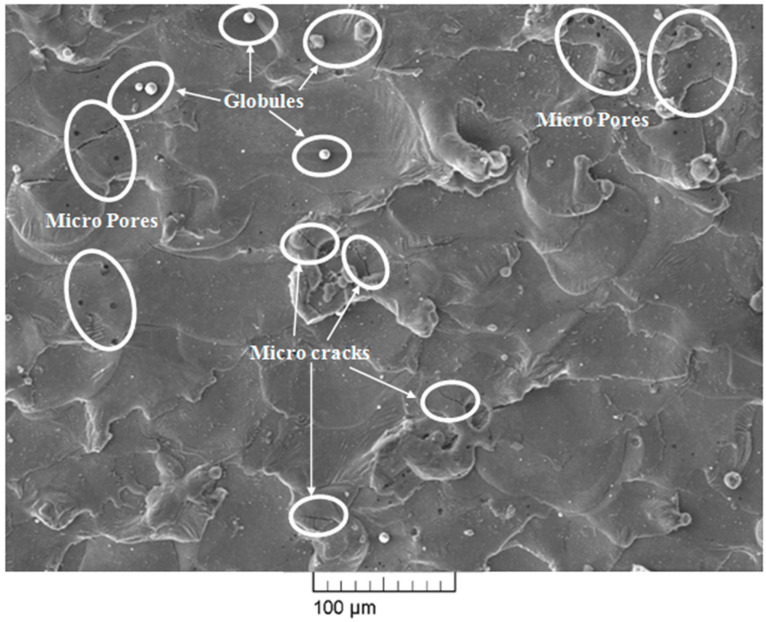
SEM micrograph at high discharge energy level.

**Figure 9:**
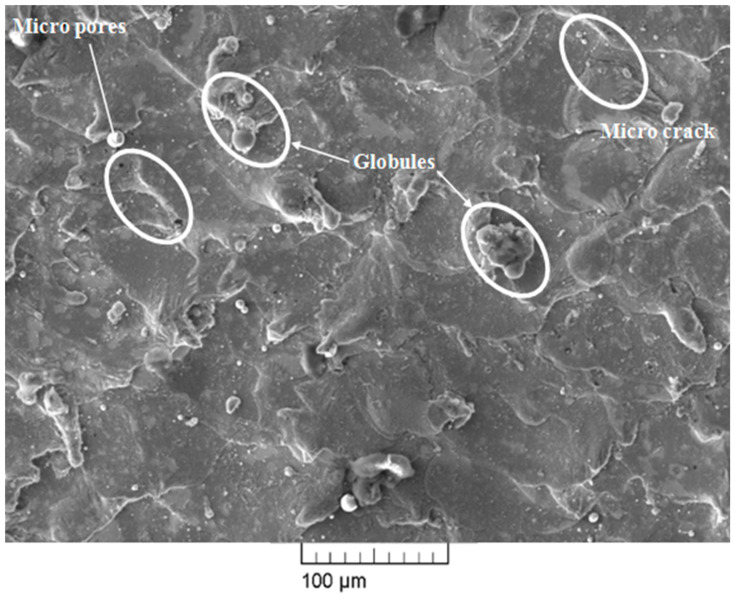
SEM micrograph at optimized parameter settings.

SEM images of the surface machined using the WEDM process are shown in Figure 7, Figure 8 and Figure 9. The WEDM process creates a series of sparks that helps to remove the material from the work surface by melting [25]. The WEDM process consists of a high number of process parameters that should be controlled to acquire great surface quality free of microcracks, micropores, and other surface defects. Higher values of pulse-on time and current result in a higher discharge energy level due to an increase in spark intensity. This in turn increases the formation chances of microcracks, globules, micropores, and other surface defects [26,27]. However, a higher value of pulse-off time decreases the discharge energy level and thereby also decreases the formation chances of microcracks, globules, micropores, and other surface defects. The surface morphology of the machined surface at low and high discharge energies was studied. WEDM process parameter settings for high discharge energy were T_on_ of 110 μs, T_off_ of 1 μs, and current of 6 A, whereas parameters for low discharge energy were T_on_ of 1 μs, T_off_ of 32 μs, and current of 1 A. Figure 7 and Figure 8 show the SEM micrograph of the machined surface at low and high discharge energy levels, respectively. It is clearly evident from Figure 7 and Figure 8 that microcracks, micropores, and deposited layers formed more readily and globule sizes were increased at the high discharge energy level in comparison with the surface morphology observed at the low discharge energy level. At the high discharge energy level, high temperature melts and evaporates the material, and then it gets mixed with the dielectric fluid. The material is then quenched in the dielectric fluid as its temperature gets reduced after mixing with the dielectric fluid. This quenching phenomenon results in the formation of cracks, pores, and other defects [28]. In addition to this, high discharge energy increases the material removal rate. Due to this excess material removal, some of the debris particles stick in the working zone and get redeposited on the machined surface. However, quenching and material deposition phenomena are markedly reduced at the WEDM parametric setting of low discharge energy when compared to those observed at high discharge energy. Due to this reason, Figure 7 shows little presence of microcracks, micropores, and deposited layers and small globules. However, the complete elimination of microcracks, micropores, deposited layers, and globules is very difficult as there will be some amount of discharge energy at all times at any parameter settings of the WEDM process. This shows that lower discharge energy is best suited for improving the surface integrity of the machined surface. However, WEDM parameters at the lowest discharge energy will not be able to satisfy all the objectives. Achieving higher MRR, T_on_, and current requires using a higher discharge energy level while keeping T_off_ at a lower level. Obtaining lower SR, T_on_, and current requires using a lower energy discharge level while keeping T_off_ at a higher level. This conflicting situation can be efficiently tackled by optimizing the WEDM process parameters for multiple responses. The WEDM process requires the optimized parameter settings of discharge current, T_on_, T_off_, and dielectric fluid for machining of nitinol SMA [15]. Our previous study of parametric optimization using the heat transfer search (HTS) algorithm proved to be highly effective in predicting and optimizing the WEDM process parameters [21]. WEDM process parameter settings at optimized conditions are T_on_ of 40 μs, T_off_ of 12 μs, and current of 1 A. Obtained values at optimized conditions show that the alloy has low discharge energy but not the lowest within the available machining range. These optimized parametric settings have been considered for the analysis as they can satisfy multiple objectives simultaneously, along with being suitable for surface analysis; more importantly, they allow the alloy to retain the shape memory effect even after machining. Figure 9 shows the SEM micrograph of the surface machined under optimized parameter settings. Little presence of microcracks, micropores, and globules can be observed on the machined surface. However, these surface defects are minimized in comparison with our previously reported study of the surface analysis of superelastic SMA [22]. The reason for this is the lower amount of discharge energy under the optimized conditions of the current study. Table 3 shows the comparison of the present study with our previously reported study in terms of SEM and EDX analysis. As discussed earlier, the complete elimination of these defects is very difficult as there will be some amount of discharge energy at all times at all parameter settings for the WEDM process. However, these defects are very small as compared to the defects obtained at a high discharge energy level. Thus, a reduction in deterioration of the machined surface can be observed to a large extent under the optimized parameter settings in the present study. 

**Figure 10:**
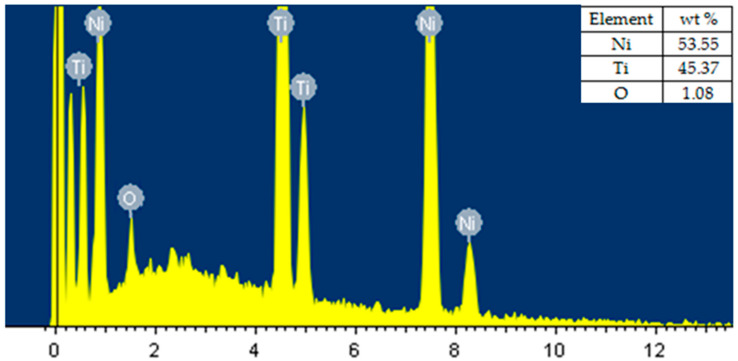
EDX analysis at optimized process parameters.

Results of the energy-dispersive X-ray analysis (EDX) of the surface machined under optimized parameter settings are shown in Figure 10. Figure 10 was also captured at the same location as Figure 9 was captured, i.e., at the center of the work sample. The chemical component values presented were obtained by using EDX analysis on SEM, which provides exact values. Moreover, the instrument was calibrated before the testing. The presence of wire material has been observed on the machined surface in past studies while using brass wire as the tool electrode [29,30]. In the current study, reusable molybdenum (Mo) wire was used as the tool electrode while machining nitinol SMA. All the important elements such as Ti, Ni, O, C, Al, Cu, and Mo were taken into consideration during the EDX analysis of the machined surface. A small amount of oxygen and high Ni and Ti contents were found in the results of EDX analysis. High activity of Ni and Ti atoms and higher temperature in the circuit are the main reasons for the presence of oxygen content [31]. Figure 10 shows the work surface to be free of Mo. The selected parameters were able to realize machining of the alloy without deposition of wire material on the work surface. The melting point of molybdenum is very high, and it has fewer tendencies to react chemically [32]. This in turn reduces the chances of diffusion of material between the molybdenum wire and workpiece [33]. Due to this reason, the wire material was not deposited on the work surface. Thus, this shows the suitability of the tool electrode for the WEDM of nitinol SMA. The novelty of the results obtained under the optimized parameter settings lies in the demonstrated capabilities such as simultaneous optimization of multiple response variables, retention of shape memory effect, improvement in surface integrity, and surface free of molybdenum (tool electrode). The present study provides significant input to end-users for the selection of WEDM process parameters for nitinol SMA.

## 4. Conclusions

In the current study, 3D surface analysis and SEM and EDX analyses were performed to improve the surface integrity of the machined samples after the WEDM process. The influence of WEDM parameters (T_on_, T_off_, and current) on the surface integrity of nitinol was studied at optimized parameters. 3D surface analysis of the machined surface depicted the higher value of SR at the top portion due to the side-flushing mechanism. However, lower SR was observed at the bottom portion of the machined surface because of the lower exposure of this area to the dielectric fluid. Thus, it is recommended to have identical flushing pressure coming from both directions to avoid any variation in SR value on the machined surface. The surface morphology of the surface machined under optimized parameter settings revealed the reduction of microcracks, micropores, and globules in comparison with morphology obtained at a high discharge energy level. A small amount of oxygen and high Ni and Ti contents were found in the results of the EDX analysis. EDX analysis showed the absence of Mo (wire material) on the machined sample. Thus, the selected set of parameters (T_on_ of 40 μs, T_off_ of 12 μs, and current of 1 A) and tool electrode (Mo) are preferable for the WEDM process of nitinol SMA as they are capable of optimizing multiple responses, retaining the shape memory effect, reducing surface defects, and obtaining a Mo-free work surface. 

## Figures and Tables

**Table 1:** Chemical composition of nitinol shape memory alloy (SMA).

Element	Ti	Ni	Co	Cu	Cr	Fe	Nb	C	H	O	N
Wt (%)	Balance	55.78	0.005	0.005	0.005	0.012	0.005	0.039	0.001	0.034	0.001

**Table 2:** 3D surface analysis comparison of present study with past study [22].

Researcher	Work Material	Input Parameters at High Discharge Energy Level	Input Parameters at Low Discharge Energy Level	Input Parameters at Optimized Parameters Settings	Remarks
Chaudhari et al. [22]	Superelastic Nitinol SMA	T_on_ = 110 μs, T_off_ = 32 μs, Current = 6 A	Not reported	T_on_ = 65 μs, T_off_ = 32 μs, Current = 6 A	3D surface analysis shows higher value of SR (1 to 148 µm) on machined surface at optimized parameter settings (Figure 4).
Present study	Nitinol SMA	T_on_ = 110 μs, T_off_ = 1 μs, Current = 6 A	T_on_ = 1 μs, T_off_ = 32 μs, Current = 1 A	T_on_ = 40 μs, T_off_ = 12 μs, Current = 1 A	Figure 3 of 3D surface analysis shows lower value of SR (1 to 75 µm approx.) on machined surface at optimized parameter settings. This is due to less discharge energy at optimized parameter settings.

**Table 3:** SEM and EDX analysis comparison of present study with past study.

Researcher	Work Material	Input Parameters at High Discharge Energy Level	Input Parameters at Low Discharge Energy Level	Input Parameters at Optimized Parameters Settings	Remarks
Chaudhari et al. [22]	Superelastic Nitinol SMA	T_on_ = 110 μs, T_off_ = 32 μs, Current = 6 A	Not reported	T_on_ = 65 μs, T_off_ = 32 μs, Current = 6 A	SEM analysis showed a large number of microcracks, micropores, and globules at a high discharge energy level, and they were largely reduced at optimized parameter settings. EDX analysis showed the presence of nickel and titanium elements of the workpiece and little amount of oxygen. Work surface was observed to be without the deposition of wire material.
Present study	Nitinol SMA	T_on_ = 110 μs, T_off_ = 1 μs, Current = 6 A	T_on_ = 1 μs, T_off_ = 32 μs, Current = 1 A	T_on_ = 40 μs, T_off_ = 12 μs, Current = 1 A	Figure 8 of SEM analysis shows some larger cracks as compared to the past study due to a slightly higher discharge energy level due to a decrease in the value of T_off_. However, reduction in other defects can be seen in Figure 9 at optimized parameter settings. Similar to past study, no wire material was found on the work surface, and the amount of oxygen was also reduced to 1.08% by wt. This is again due to less discharge energy in comparison to past results.
